# Current and Prospective Protein Biomarkers of Lung Cancer

**DOI:** 10.3390/cancers9110155

**Published:** 2017-11-13

**Authors:** Tatiana N. Zamay, Galina S. Zamay, Olga S. Kolovskaya, Ruslan A. Zukov, Marina M. Petrova, Ana Gargaun, Maxim V. Berezovski, Anna S. Kichkailo

**Affiliations:** 1 Laboratory for Biomolecular and Medical Technologies, Krasnoyarsk State Medical University named after prof. V.F. Voino-Yaseneckii, 1 P. Zheleznyaka, Krasnoyarsk 660022, Russia; tzamay@yandex.ru (T.N.Z.); galina.zamay@gmail.com (G.S.Z.); olga.kolovskaya@gmail.com (O.S.K.); 2 Departments of Physiology, Krasnoyarsk State Medical University named after prof. V.F. Voino-Yaseneckii, 1 P. Zheleznyaka, Krasnoyarsk 660022, Russia; 3 Federal Research Center, Siberian Branch of the Russian Academy of Science, 50/24 Akademgorodok, Krasnoyarsk 660036, Russia; 4 Departments of Oncology and Radiation Theraphy, Krasnoyarsk State Medical University named after prof. V.F. Voino-Yaseneckii, 1 P. Zheleznyaka, Krasnoyarsk 660022, Russia; zukov_rus@mail.ru; 5 Krasnoyarsk Regional Clinical Cancer Center named after A.I. Kryzhanovsky, Krasnoyarsk 660133, Russia; 6 Departments of Polyclinic therapy and Family Medicine, Krasnoyarsk State Medical University named after prof. V.F. Voino-Yaseneckii, 1 P. Zheleznyaka, Krasnoyarsk 660022, Russia; stk99@yandex.ru; 7 Independent Researcher Vancouver, 207-2145 York Avenue, Vancouver, BC V6K 1C4, Canada; ana.gargaun@gmail.com; 8 Department of Chemistry and Biomolecular Sciences, University of Ottawa, Ottawa, ON K1N6N5, Canada; maxim.berezovski@uottawa.ca; 9 Departments of Pharmacology, Krasnoyarsk State Medical University named after prof. V.F. Voino-Yaseneckii, 1 P. Zheleznyaka, Krasnoyarsk 660022, Russia

**Keywords:** lung cancer, biomarker, histological type, aptamers, diagnostics, targeted therapy

## Abstract

Lung cancer is a malignant lung tumor with various histological variants that arise from different cell types, such as bronchial epithelium, bronchioles, alveoli, or bronchial mucous glands. The clinical course and treatment efficacy of lung cancer depends on the histological variant of the tumor. Therefore, accurate identification of the histological type of cancer and respective protein biomarkers is crucial for adequate therapy. Due to the great diversity in the molecular-biological features of lung cancer histological types, detection is impossible without knowledge of the nature and origin of malignant cells, which release certain protein biomarkers into the bloodstream. To date, different panels of biomarkers are used for screening. Unfortunately, a uniform serum biomarker composition capable of distinguishing lung cancer types is yet to be discovered. As such, histological analyses of tumor biopsies and immunohistochemistry are the most frequently used methods for establishing correct diagnoses. Here, we discuss the recent advances in conventional and prospective aptamer based strategies for biomarker discovery. Aptamers like artificial antibodies can serve as molecular recognition elements for isolation detection and search of novel tumor-associated markers. Here we will describe how these small synthetic single stranded oligonucleotides can be used for lung cancer biomarker discovery and utilized for accurate diagnosis and targeted therapy. Furthermore, we describe the most frequently used in-clinic and novel lung cancer biomarkers, which suggest to have the ability of differentiating between histological types of lung cancer and defining metastasis rate.

## 1. Introduction

Lung cancer is the most common cancer and the leading cause of cancer-related deaths worldwide, with approximately 1.8 million new cases and 1.6 million deaths in 2012 [1,2,3,4]. The five-year survival rate of patients with lung cancer is approximately 13–15% [5]. Diagnosis of lung cancer at late stages is a determining factor of high mortality from the disease. It is primarily explained by metastases from lung into the central nervous system as observed in 54% cases [6,7]. Therefore, early lung cancer detection is necessary for reducing the high mortality rate. Understanding the biological mechanisms of tumor development and biomarker expression typical for lung cancer and specific for all histological types is crucial for accurate diagnosis, treatment, and drug development. 

## 2. Histology of Lung Cancer Types

**Figure 1:**
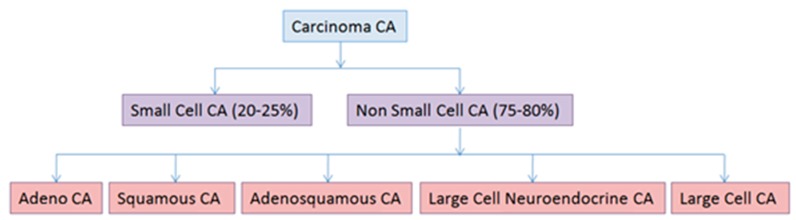
The new World Health Organization (WHO) classification of lung cancer histological types. The various types of lung cancer have different origins and histological features (Figure 2). Small-cell lung carcinoma (SCLC) is characterized by small size cells, absence of differentiation, fast tumor growth, metastasis at early stages, and release of specific biomarkers and hormones. At present, there are two points of view on SCLC histogenesis. According to the first hypothesis, SCLC arises from cells of the diffuse endocrine system, i.e., the amine precursor uptake decarboxylation (APUD)-system (Figure 2); the second suggests this type of lung cancer originates from the endodermbronchial lining layer [10]. CA: carcinoma.

Lung cancer is a malignant lung tumor that may stem from bronchial epithelium, bronchioles, alveoli, and bronchial mucous glands. It is characterized by post-treatment relapses, metastasis, and a variety of histological types. In 2015, the new World Health Organization (WHO) classification of lung tumors was published [8]. The two main lung cancer types are small-cell lung carcinoma (SCLC) and non-small-cell lung carcinoma (NSCLC) (Figure 1). Approximately 80% of lung cancer cases are NSCLC, which have diverse molecular-biological features and clinical course forms of the disease [8,9]: adenocarcinoma, adenosquamous carcinoma, squamous cell carcinoma, large cell carcinoma, and large cell neuroendocrine carcinoma.

**Figure 2:**
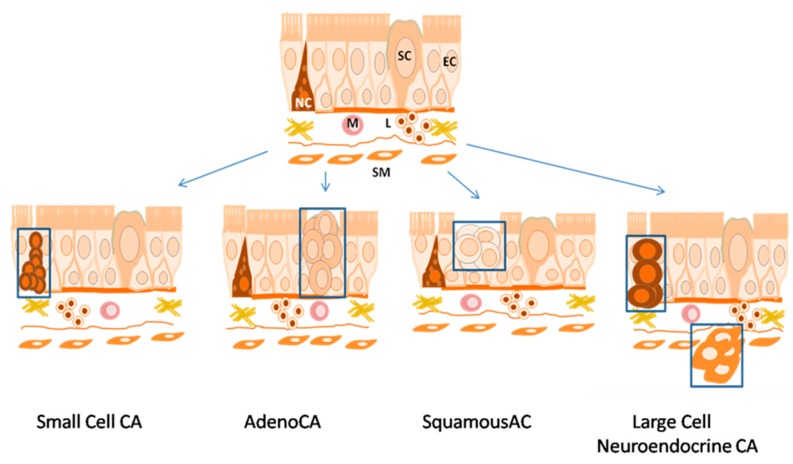
Histogenesis of histological types of lung cancer. SM—Smooth Muscle; M—Macrophage; L—Lymphocyte; NC—Neuroendocrine Cell; EC—Epithelial Cell; SC—Secretory Cell.

Among the other subtypes of NSCLC, adenocarcinoma arises from glandular cells of bronchial mucosa and now represents the dominant histological subtype among the other lung cancer types (Figure 2). Squamous lung cancer arises from the modified bronchial epithelial cells (Figure 2) and is characterized by one of the following specific differentiation features: keratinization, keratin pearl formation, or the presence of intercellular bridges. Аdenosquamous carcinoma is a type of cancer that contains two types of cells: squamous cells (thin, flat cells that line certain organs) and gland-like cells [9]. Large cell neuroendocrine carcinoma is a malignant epithelial tumor, which is comprised of large polygonal cells that do not show any obvious evidence of histological differentiation. The cases include large cell neuroendocrine carcinoma, basaloid carcinoma, lymphoepithelioma-like carcinoma, and clear cell carcinoma. The tumor arises from neuroendocrine cells of the respiratory tract lining layer or smooth muscle cells of its wall (Figure 2). Large-cell carcinoma is a heterogeneous group of undifferentiated malignant neoplasms that lack the cytologic and architectural features of small cell carcinoma and glandular or squamous differentiation. Large-cell carcinoma is categorized as a subtype of NSCLC that originates from epithelial cells of the lung [11].

It is well known that a unique combination of exogenous and endogenous factors influences the occurrence and development of lung cancer in each individual. Therefore, lung cancer, like other oncological diseases, is heterogeneous. Thus, in addition to various histological types, this disease also has many molecular and pathological subtypes characterized by heterogeneous cellular genetic and epigenetic changes and a different combination of protein biomarkers. However, at present, data on protein signatures of molecular subtypes of histological types of lung cancer is extremely limited, but a large number of genetic studies reflecting the probability of certain mutations in genes are presented. In particular, mutations of EGFR (epidermal growth factor receptor) in lung adenocarcinoma have been well studied. It was found that in patients with lung adenocarcinoma, the probability of EGFR mutations increases linearly from age 3.7% (18–30 years) to 18.5% (81–100 years), and in female non-smokers, the probability of mutations is higher than in men [12,13]. In male non-smokers, the probability of EGFR mutation is much higher than in smokers [12].

Identification of the correct histological type of lung cancer and their molecular subtypes is necessary due to different treatment strategies. Tumor cells of each histological type release certain protein biomarkers into the bloodstream and therefore play a key role in cancerogenesis. The use of blood plasma to determine the origin and nature of the malignant cells for diagnosis requires knowledge about expression of protein biomarkers, their specificity, sensitivity, and their release by different types of lung cancer cells [14,15,16]. 

## 3. Methods Currently Employed to Diagnose Lung Cancer

Currently, lung cancer is detected mostly in the late stages due to such symptoms as coughing, coughing up blood, shortness of breath, and chest pains. Unfortunately, the early stages of this disease are often detected only by accident. Chest radiography and computer tomography are the most commonly used methods for lung cancer diagnosis. However, as they can only identify visible and irreversible changes in lung, there is a need for additional methods for early diagnosis. In order to overcome this challenge, it is essential to discover novel, highly sensitive, and specific biomarkers [15].

## 4. Circulating Biomarkers of Carcinogenesis

Diagnostic significance of protein biomarkers is defined by their sensitivity and specificity. Biomarker sensitivity is determined by the percentage of true positive results of analysis in a group of oncological patients, while biomarker specificity is determined by a percentage of true negative results of analysis in a group of healthy people and patients with benign diseases. Unfortunately, to date, 100% sensitive and specific biomarkers have not been found. Moreover, some cancer-specific biomarkers were also found in plasma of healthy people.

For non-invasive detection of lung cancer biomarkers, biological materials such as tumor tissues, blood, exhaled breath condensate, sputum, and urine are usually utilized. Exhaled breath condensate is liquid received from the respiratory tract that consists of cytokines, proteins, and DNA [17]. It has been established that the content of condensate from lung cancer patients differs from condensate of healthy people, nevertheless, specific protein biomarkers have not been detected. Similarly, in sputum, one of the most attractive non-invasive source of lung cancer biomarkers, specific markers are also yet to be detected [18].

Thus, blood remains the most perspective source for biomarker discovery because cellular debris penetrate into the bloodstream from the tumor. As a result, blood may be used as a minimally invasive liquid biopsy. Blood is a complex matrix containing tumor-associated circulating lipids, proteins, RNAs, miRNAs, DNAs, as well as cancer, immune, stromal, and endothelial cells [19].

Tumor-associated biomarkers are biological molecules that can be detected and serve as indicators of pathogenic processes or pharmacological/pharmacodynamic response to treatment [20]. Different oncomarkers can be used to distinguish normal and pathogenic processes [4]. An ideal biomarker originates from neoplastic cells, is indiscernible in healthy and benign tissues, and can be identified by simple methods in the available biological material (biological fluids). It should be sensitive, specific and cost-saving. 

Tumor biomarkers are divided into several types: genetic (mutations, changes in number of copies, matrix RNA expression), epigenetic (changes in DNA methylation profile), proteomic (changes in level and profile of protein expression), metabolic (changes in level and spectrum of low molecular weight metabolites), DNAs and RNAs circulating in blood plasma, exosomal microRNAs (miRNAs), synthesis profile and level of miRNAs, protein biomarkers, circulating tumor cells (CTCs), and immune, stromal, and endothelial cells [17,18,20,21,22,23,24,25,26]. Overall, proteins are the most suitable biomarkers for lung cancer diagnosis because of their involvement in cellular processes. A panel of biomarkers (in particular, CYFRA 21-1 (cytokeratins), EPCAM (epithelial cell adhesion molecule), ProGRP (pro-gastrin-releasing peptide), CEACAM (carcinoembryonic antigen), and others are used for screening various malignancies including lung cancer. However, in practice, this system often fails in providing sufficient sensitivity and information of value for optimal screening. For example, for the diagnosis of lung cancer, CEACAM sensitivity and specificity is 69% and 68% respectively, while that for CYFRA 21-1 was 43% and 89%, respectively [27]. Diagnostic sensitivity of plasma ProGRP in distinguishing SCLC was estimated to be approximately 84%, and specificity 95% [28]. For comparison, CT has a sensitivity is about 94% with low specificity and high false-positive rate in the detection of lung cancers [29].

Various approaches are used for biomarker discovery; in particular, mass spectrometry analysis is commonly used for protein profiling of tumors and another popular method, which will be discussed, is affinity-based enrichment using aptamers and other molecules.

## 5. Proteomic-Based Lung Cancer Biomarker Search

Mass spectrometry methods allow identification and analysis of thousands of proteins in biological systems [30]. Mass spectrometry can provide valuable information such as the differences between protein profiles of normal and tumor lung tissue. As such, Kang and coauthors established that the main lung cancer biomarker is the β-chain of human HP (haptoglobin) [31], others also classified SAA (serum amyloid A) [4], APOA1 (apolipoprotein A-1) [32], ANXA (annexin), VIM (vimentin), NM (non-muscle myosin), CALM (calmodulin), CFL (cofilin),TMS (thymosin), and EGFR (epidermal growth factor receptor) as lung cancer biomarkers [30] (Table 1).

Mass spectrometry helped discriminate the differences between protein profiles of adenocarcinoma and squamous lung cancer. Nevertheless, despite numerous known tumor-associated biomarkers, sensitivity of proteomic studies is insufficient—only 79% for the first and second stage of lung cancer [49]. Low sensitivity is most likely related to the loss of a number of low copy proteins which are present in tissues and blood in trace amount. 

## 6. Protein Biomarkers Used in Lung Cancer Diagnosis

Early diagnosis of lung cancer could be based on detection of protein markers and autoantibodies specific for each type of cancer [50]. In particular, screening studies conducted in the United States where 1613 patients used EarlyCDT^®^-Lung test revealed lung cancer at stage I. The blood test detected early lung cancer in asymptomatic patients with higher specificity than imaging tests [50]. A similar level of specificity and sensitivity for lung cancer diagnosis using autoantibodies was achieved in the studies conducted by Caroline J. Chapman [51]. Thus, high sensitivity and specificity levels make the panel of autoantibodies an important addition to the standard methods for early diagnosis of lung cancer.

Despite the great advances made in lung cancer biomarker discovery, no data on biomarkers with high enough specificity and sensitivity has been found. This is related to a number of reasons: (1) inefficiency of techniques applied for biomarker search, (2) genetic heterogeneity of tumors, (3) poor reproducibility of laboratory tests, (4) poor research design, (5) low concentration of analyzed biomarkers, and (6) insufficient number of tissue banks for screening [25].

Nevertheless, lung cancer therapy has advanced mostly due to target treatment approaches. However, it was found that effective therapy for each histological type of lung cancer requires prior knowledge about specific molecular targets. This establishes the importance of both the diagnosis of lung cancer as well as further identification of the histological type. Several biomarkers are currently used for clinical lung cancer detection. However, a variety of proteins that may act as novel tumor-associated markers are yet to be proven (Table 2).

Some clinically used biomarkers such as CEACAM, CYFRA 21-1, and ProGRP have low concentrations in serum, and therefore, each biomarker alone cannot be used for early lung cancer diagnosis [52,53]. As such, they are used as a mixture. In particular, the combination of CEACAM and CYFRA 21-1 could be used for detection of adenocarcinoma [54]. Statistically significant differences between lung cancer patients and healthy people were found using a panel of CEACAM, CA125, CYFRA 21-1, and NY-ESO (cancer-testis antigen) [45]. Other authors also used NSE (neuron specific enolase), CEACAM, and CYFRA 21-1 for differentiation of histological subtypes of lung cancer [29].

The use of serum markers, namely LDH (lactate dehydrogenase), CRP (C-reactive protein), CEACAM, NSE, and CYFRA 21-1, has enhanced accuracy of lung cancer diagnosis to 94.8% [42]. William L. Bigbee and coauthors suggest a panel of biomarkers—PRL (prolactin), TTR (transthyretin), THBS1 (thrombospondin 1), SELE (selectin E), MIF (macrophage migration inhibitory factor), PLAT (plasminogen activator, tissue type), EGFR, ERBB2 (erb-b2 receptor tyrosine kinase 2), CYFRA 21-1, and serum APBA (2-dehydropantoate 2-reductase)for early lung cancer diagnosis with 77.1% sensitivity and 76.2% specificity [80]. The combination of CEACAM, RBP (retinol-binding proteins), SERPIN (serpin peptidase inhibitors), and SART (U4/U6.U5 tri-snRNP-associated protein 1)was also used to diagnose lung cancer and classify patients in the independent validation set (sensitivity—77.8%; specificity—75.4%) [81].

An overview of recent publications on early lung cancer diagnosis showed that combinations of a variety of tumor-associated biomarkers could be more useful than using each of them separately [82]. Nevertheless, no composition for detection of lung cancer at early or premalignant stages has been found [83].

## 7. Aptamer-Based Affinity Enrichment Methods for Lung Cancer Biomarker Discovery

In recent years, a new specific technique of biomarker discovery using aptamers, i.e., AptaBID, has been developed [64]. Aptamers are small single-stranded DNA or RNA (30–100 nt) oligonucleotides that form three-dimensional structures capable of binding certain targets. Specific binding of the aptamers is conditioned by the dimensional structure, spatial charges distribution, phosphates and the mismatch of bases, capable of electrostatic and van der Waals interactions and forming hydrogen bonds [84]. Being highly selective, aptamers can quickly distinguish small differences in thousands of proteins and therefore can be used in a wide variety of applications including molecular imaging, drug delivery, therapy, diagnostics and biomarker discovery.

**Figure 3:**
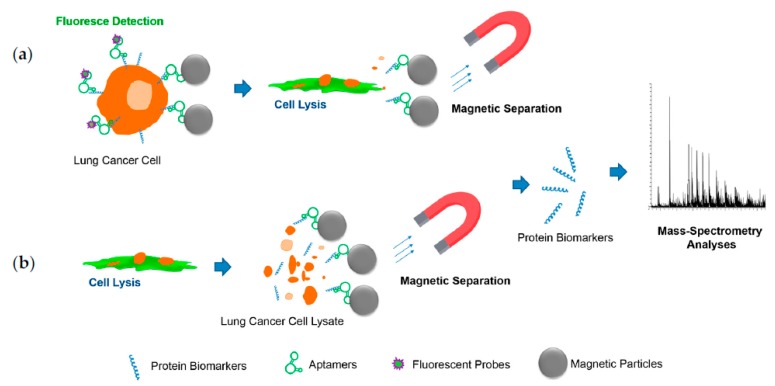
Schematic representation of aptamer based biomarker discovery. Affinity purification of aptamer protein targets: (**a**) from whole cells; (**b**) form cell lysates.

Despite the promising qualities aptamers possess for diagnostic and therapeutic purposes, their widespread application is still limited due to problems and pitfalls that currently plague the technology [85,86]. Conventional SELEX (Systematic Evolution of Ligands by EXponential Enrichment) selection and its various modifications are time and labor consuming, and no universal and automatic aptamer generation procedure has been established [85]. The main challenge is generation of efficient aptamers for in vivo applications with high affinity to viable cells and tissues, long-lasting stability in bloodstream, high selectivity, and low cross-reactivity. These “bottlenecks” could be overcome by restricting selection to in vivo like conditions and increasing the chemical diversity of oligonucleotides by addition of modified bases and chemical modifications, which introduce new functionalities to aptamers. Nucleotide chemical modification can also help prevent aptamer degradation and excretion from the bloodstream by renal filtration, increase circulation time, improve aptamer binding, and potentially expand their use for therapy and diagnostics [85,86,87]. Current selection processes became more efficient due to novel bio-separation technologies and high-throughput screening technologies. Often, aptamers generated against purified or recombinant proteins do not show good binding in vivo and sometimes have cross-reactivity [86,87]. Aptamers for diagnostic and therapeutic applications are more effective when selected against complex targets (cells, viruses, bacteria, etc.), but identification of their exact binding partner is often complicated and expensive. Specific biomarkers are isolated by aptamer-mediated affinity purification using magnetic separation from whole cells (Figure 3a) or cell lysates (Figure 3b). Purified proteins are identified using mass-spectrometry analyses (Figure 3).

Modifications of AptaBID approach were applied for aptamer facilitated detection of several lung cancer protein biomarkers such as CTSD (cathepsin D) [61], VIM (vimentin), DEF (defensin) [62,88], ANXA2 (annexin A2), ANXA5 (annexin A5), H2B (H2B histone family member M), and CLU(clusterin) [62], LMN (lamin), and TUB (tubulin), ACT (actin) [88]. 

CTSD is implicated in tumorigenesis and is hyper-expressed in lung cancer tissues and plasma [89]. ANX (annexin) are considered as targets of breast cancer, pancreatic cancer and laryngeal carcinoma therapy alone and/or synergistically [90]. Ubiquitylated H2B in cancer cells plays an important role in human malignancy [91]. VIM, a major constituent of the intermediate filament family of proteins is involved in cancer initiation and progression, tumorigenesis, metastasis formation, and epithelial-to-mesenchymal transition [92,93,94]. Its expression is increased in moderately and well-differentiated adenocarcinoma and in giant cell carcinoma [92,95]. LMN of the nuclear lamina modulate cell proliferation, differentiation, as well as epithelial-mesenchymal transition and migration [96,97]. It serves as a marker of good or poor patient survival depending on tumor subtype [98,99]. TUB hyperexpression and their post-translational modifications are correlated with poor prognosis and chemotherapy resistance of various cancers [100]. DEF through EGFR activation and downstream signaling pathways, influence cell migration and proliferation [101] and are associated with cancer invasiveness [102].

Some proteins expressed by circulating tumor cells (CTC) have lung origin, and thus, aptamers for these markers could be used to isolate specific CTC from blood [62]. Some of these cancer-related targets were found in crude blood plasma of patients with NSCLC and SCLC and could be detected with an electrochemical aptamer–based sensor [103]. Lung cancer cells express the same biomarkers, and therefore corresponding aptamers could be used for histological structure characterization of lung adenocarcinoma. The selected DNA aptamers showed binding to various tumor structures, such as elastic fibers, tumor cells, blood vessels, and elastin, which play important roles in tumor formation and growth [88].

Another powerful aptamer-based proteomic technology allowing large-scale comparison of proteome profiles in small volumes of biological samples with low limits of detection, a broad dynamic range, and high reproducibility has been suggested by Larry Gold and SomaLogic [104]. This approach enables the discovery of novel biomarkers using Slow Off-rate Modified Aptamers (SOMAmers) for affinity enrichment [104]. SOMAmers engage proteins and increase the range of epitopes available for binding because of their more hydrophobic surfaces compared with conventional aptamers [82]. 

A highly multiplexed proteomic technology SOMAscan technology demonstrated the possibility of sensitive proteomic assay of protein expression signatures in NSCLC using healthy adjacent and distant tissues from surgical resections [82]. Thirty-six proteins with the largest mean fold-change in protein expression between tumor and non-tumor tissue samples have been suggested as novel protein biomarkers of NSCLC and were classified into biological processes associated with important hallmarks of cancerogenesis: angiogenesis, growth and metabolism, inflammation and apoptosis, and invasion and metastasis [82]. 

## 8. Lung Cancer Diagnosis Using Aptamers

Сancer-related proteins can be detected using various sensors, most of them rely on antibody-antigen interaction in a sandwich-like system that requires two different types of antibodies for target identification. The main challenge in the development of reliable diagnostic sensors based on antibodies is due to the fluctuation of their affinity depending on manufacture and batch. Synthetic aptamers overcome these limitations as their properties at the same conditions depend only on nucleotide sequence [105,106]. Another advantage of using aptamers over the conventional antibodies is the possibility to modify them chemically with various labels, active groups, and nanoparticles, which is important for biosensors design [105]. Some aptamers change their conformation after binding to their target molecules, which makes the development of switchable aptasensors and fluorescence quenching sensors possible, which could not be achieved with antibodies [107]. Thus, aptamers are widely used for development of various diagnostic tools (optical, colorimetric, fluorescence, electrochemical, microfluidic, PET (positron-emission tomography), CT (computed tomography), NMR (nuclear magnetic resonance), MRI (magnetic resonance imaging), or ultrasound imaging etc.) [105,106,107]. 

**Figure 4:**
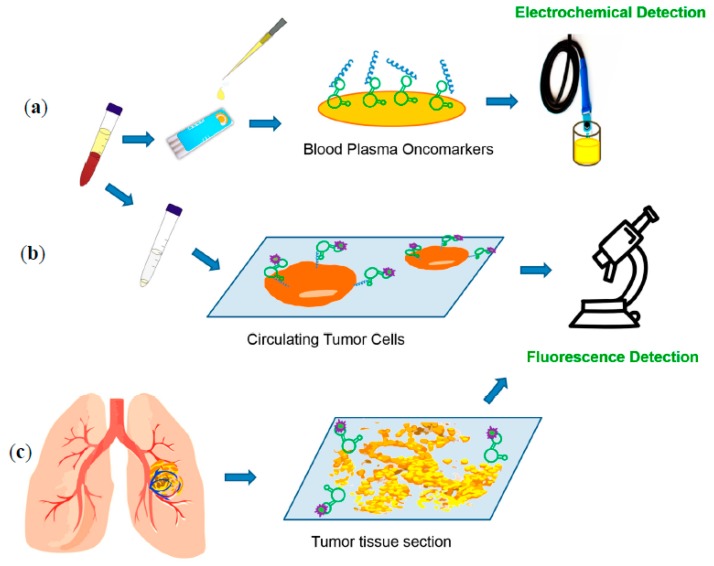
Schematic representation of aptamer-based lung cancer diagnostic tools. (**a**) analyses of blood plasma oncomarkers using electrochemical detection; (**b**) circulating tumor cells capture and fluorescence detection ; (**c**) aptamer based immunohistochemistry-like characterization of lung cancer histological structure.

More than 20 different aptamers selected by a number of research groups all over the world demonstrated their high sensitivity for lung cancer diagnosis in different sensor systems as well as a unique potential for a targeted therapy (Table 3, Figure 4). 

Aptamers selected to postoperative adenocarcinoma tissues have been utilized for detection of circulating tumor cells in blood [62], characterization of histological structure of lung adenocarcinoma [88], and electrochemical sensing of blood plasma biomarkers [103].

Aptamers to lung cancer were developed not only for diagnostics, but also for cancer treatment. Aptamers alone demonstrate antitumor activity in cell cultures; S13, S50 inhibit proliferation [111], LC-183 suppress cancer cell growth [113], and R50 cause apoptosis [112]. Aptamers are effective targeting ligands; anti mucin-1 aptamer is suitable for carrying doxorubicin [114] and plasmid DNA [115] to cancer cells in most adenocarcinomas. Aptamer GL21.T are used as carriers for selective delivery of a miRNA to A 549 cells, processing by the RNA interference machinery, and silencing let-7 g target genes, thus suppressing let-7 g function. This conjugate reduced tumor growth in vivo in a xenograft lung adenocarcinoma model [116]. Aptamers to NCL (nucleolin) have been used for targeted delivery of siRNA chimeras for lung cancer therapy [118] and PET imaging in vivo in a xenograft lung adenocarcinoma model [119]. 

Several aptamers against SCLC cells have high affinity and specificity in different assay formats for cell lines and tissues from the patient samples. Conjugates of these aptamers with magnetic and fluorescent nanoparticles effectively extracted SCLC cells from mixed cell media for isolation, enrichment, and sensitive detection [108]. Other aptamers to SCLC SBC3 cell line with good selectivity are suitable for fluorescence microscopy and flow cytometry analyses. Unfortunately, their exact protein targets have not yet been determined. 

### Aptahistochemistry for Identification of Lung Cancer Biomarkers

An important clinical method often used for diagnosis of tumor biomarkers is immunohistochemistry. A commonly used marker to identify adenocarcinoma is TTF-1 (transcription termination factor 1) [121], but in 70–90% cases of small cell lung cancer, expression of this marker is present. Squamous cell carcinoma biomarkers such as TP63 (tumor protein p63), CK5/6 (cytokeratin 5/6), 34βE12 (high molecular weight cytokeratins) could also be identified in adenocarcinoma [121]. Immunohistochemistry of small cell lung cancer is required only in problematic cases; usually, hematoxilin and eosin staining is sufficient for diagnosis. AE1/AE3 (pancytokeratin) is used to demonstrate that the tumor is a carcinoma rather than a lymphoid lesion [9]. 

Immunostaining is based on histological identification of tumor biomarkers and abnormal blood vessels by specific agents such as antibodies, but this method has some limitations such as: relatively high cost, difficulties in quantifying results, probes in tissue immunohistochemistry-like staining, as described in the research by Galina S. Zamay et al. [88]. They have shown that DNA-aptamers previously selected to postoperative lung cancer tissue specifically bind to different structures of tumor tissue including elastic fibers, tumor cells, blood vessels and elastin, having an important role in the formation of tumors. Protein binding partners of the aptamers were identified using affinity purification followed by mass spectrometry analyses, and validated with correspondent antibodies. According to this data, LMN (lamin), VIM (vimentin), TUB (tubulin), and ACT (actin) detected with the help of aptamers LC-18, LC-17, and LC-24 are involved in cancer progression and could act as lung adenocarcinoma biomarkers [88].

## 9. Biomarkers of Different Histological Lung Cancer Types

**Figure 5:**
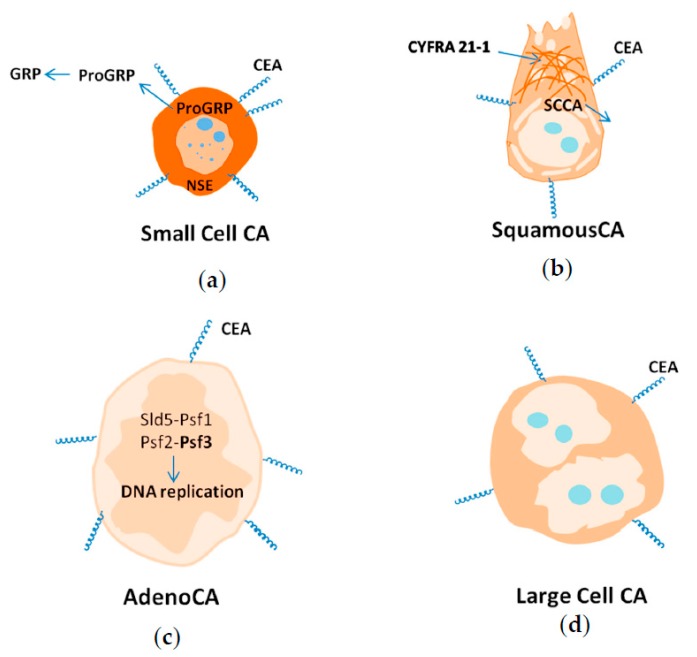
Biomarkers of Small Cell Lung Cancer (**a**), Squamous Lung Cancer (**b**), Lung Adenocarcinoma (**c**), Large Cell Lung Cancer (**d**). GRP: gastrin-releasing peptide ; CEA: carcinoembryonic antigen ; NSE: neuron specific enolase; SCCA: squamous cell carcinoma antigen ; CYFRA 21-1: cytokeratins ; Sid5 : Systemic RNA interference defective protein 5 ; Psf1-Psf3: GINS complex subunits 1-3.

In addition to the numerous biomarkers that are currently used for clinical lung cancer detection, other proteins of new tumor-associated markers and their respective roles are also investigated (Table 2). For example, CEA (carcino embryionic antigen) is a 180-kDa glycoprotein as well as a carcinoembryonic antigen of fetal embryonic development, in addition to this, it is also a biomarker as its concentration level is increased in blood of patients with all lung cancer types (Figure 5) [48,79]. CEA is involved in cell adhesion and modulation processes [57]. As a result, tumors with high expression of CEA have high metastatic potential that may be caused by cell-cell adhesion between tumor and vessels because CEA is involved in homo- and heterotypic interactions with other cells [72]. High levels of CEA in serum is also correlated with brain metastases [122,123]. Serum levels of CEA may be useful for assessment of prognostic information about the risk of recurrence and death from lung cancer [76,124]. Importantly, the level of CEA does not correlate with the stage of the disease [72].

### 9.1. Small Cell Lung Cancer

SCLC arises from neuroendocrine cells of the APUD-system (amine precursor uptake and decarboxylation system) [67] and has two of the main biological features of these cells—production of L-DOPA-decarboxylase (L-3,4-dihydroxyphenylalanine- decarboxylase) and NSE (Figure 5a). L-DOPA decarboxylase is the gene encoding for the enzyme that catalyzes the biosynthesis of dopamine in humans [125]. NSE is a glycolytic neuron specific isoenzyme of enolase with two almost identical 39-kDa polypeptides produced in the central and peripheral neurons and malignant tumors of neuroectodermal origin; NSE is specific only for SCLC [42]. Adrenocorticotropic hormone, serotonin, antidiuretic hormone, calcitonin, growth hormone, melanocyte-stimulating hormone, and estrogen are also produced in SCLC.

The other well-known biomarker of SCLC is ProGRP (pro-gastrin-releasing peptide). High levels of ProGRP were found in the blood of patients with SCLC and medullary thyroid cancer (>200 pgmL^−1^). Blood plasma of healthy people and patients with benign diseases have ProGRP concentrations of 35 pgmL^−1^ and 45–10^3^ pgmL^−1^ respectively. ProGRP has organ specificity and does not correlate with the stage of lung cancer. ProGRP is more specific than NSE; unfortunately, the use of this biomarker for further studies is complicated due to its instability and difficulty of identification. Sensitivity and specificity of ProGRP were 80% and 90%, respectively, while NSE showed fewer rates of sensitivity and specificity—64% and 43%. However, 27% of patients with SCLC had increased levels of NSE and normal levels of ProGRP [22]. According to this data, the simultaneous detection of ProGRP and NSE should improve the sensitivity and specificity of SCLC diagnosis (Figure 5a). 

### 9.2. Squamous Lung Cancer

Squamous lung cancer arises from modified bronchial epithelial cells. One of the most distinctive features of squamous lung cancer is high levels of fragmented cytokeratin CK-19 subunit—CYFRA 21-1 (Figure 5b). CK-19 is a protein component of intermediate fibers of epithelial cells [126]. The level of CYFRA 21-1 is increased during the malignization process of normal epithelial cells. CYFRA 21-1 is highly expressed in serum of patients with a metastatic form of squamous lung cancer. In contrast to this, high concentrations of CYFRA 21-1 are not typical for SCLC [48,60,127].

The other specific protein for squamous lung cancer is SCCA (squamous cell carcinoma antigen), a 48-kDa protein which is found in increased levels in squamous lung cancer [58,127,128]. SCCA is an inhibitor of serine proteases such as human CELA (chymotrypsin), CAPN1 (calpain 1), and CTSL (cathepsin L) [129]. It also inhibits apoptosis of tumor cells and stimulates invasion and metastasis [130].

### 9.3. Adenocarcinoma 

Adenocarcinoma arises from glandular cells of bronchial mucosa and expresses several protein markers (Figure 5c). 

Diagnosis of adenocarcinoma is often based on identification of molecular markers of mutations, in particular EGFR, ERCC (DNA excision repair protein), RRM 1 (ribonucleoside-diphosphate reductase), KRAS (KRAS proto-oncogene), TS (thymidylate synthetase), and EML4-Alk (anaplastic lymphoma kinase receptor tyrosine kinase) [78]. Recently, protein PSF3 (DNA replication complex GINS) has become popular as a biomarker of adenocarcinoma [75,77,131]. PSF3 is a member of the heterotetrameric complex GINS (“go-ichi-ni-san” complex, from the first letters of the Japanese numbers 5-1-2-3) comprising SLD5 (Systemic RNA interference defective protein 5), PSF1 (GINS complex subunit 1), PSF2 (GINS complex subunit 2), and PSF3 (GINS complex subunit 3). This complex associates with proteins, which in turn regulate both the initiation and the progression of DNA replication [132]. To date, an overexpression of PSF3 in adenocarcinoma has been clearly established, which leads us to conclude that its level should be higher in blood plasma. However, data on the level of PSF3 in blood has yet to be reported. In addition to these biomarkers, several novel lung adenocarcinoma-associated proteins have been found using aptamers, such as LMN (lamin) and VIM (vimentin), DEF (neutrophil defensin) and TUB (tubulin), ACT (cytoplasmic actin), CTSD (cathepsin D), CLU (clusterin), NCL (nucleolin), and MUC1 (mucin-1). According to recent studies, identification of such proteins would improve the diagnosis of adenocarcinoma. 

### 9.4. Large Cell Carcinoma

Large cell carcinoma is a malignant epithelial tumor that comprises large polygonal cells showing no obvious evidence of histological differentiation. Large cell carcinoma is characterized by small, scattered groups of large non-differentiated, polimorphic, and often dual- or multi-core cells [11]. Data on specific biomarkers of this histological type of lung cancer have not been found (Figure 5d).

### 9.5. Adenosquamous Carcinoma

Adenosquamous carcinoma is characterized by the features of squamous cell carcinoma and adenocarcinoma simultaneously. Consequently, it has a protein biomarker of both histotypes—MUC (mucin) [58].

### 9.6. Large Cell Neuroendocrine Carcinoma

Large cell neuroendocrine carcinoma (LCNEC) is extremely rare. There are difficulties related to its diagnosis and treatment. LCNEC showed overexpression of TOP SST (topoisomerasis somatostatin precursor), and ERCC1 (excision repair 1, endonuclease non-catalytic subunit) [133].

### 9.7. Protein Biomarkers to Main Histological Types of Lung Cancer 

Despite the length at which clinically used protein biomarkers have been studied, the data shows that their levels in patients’ blood with different histological types of lung cancer varies. Table 4 presents comparative levels of well-known lung cancer biomarkers in blood plasma of patients with NSCLC, SCLC, and healthy people.

Protein biomarkers of two main histological types of NSCLC, adenocarcinoma and squamous lung cancer and their respective levels in blood plasma are compared to a healthy control group and summarized in Table 5.

Thus, the analysis of clinical biomarkers has shown that the use of six of the most specific protein biomarkers will help improve early diagnosis of lung cancer and allow differentiating between lung cancer histological types. These are summarized in Table 6.

1. CEA is a biomarker specific for all lung cancer types;
2. NSE is a biomarker of NSCLC, and a marker of metastasis;
3. CYFRA21-1 is a general biomarker for screening for lung cancer, and a biomarker of squamous lung cancer in metastatic form;
4. SCCA is a biomarker of squamous lung cancer;
5. PSF3 is a biomarker of adenocarcinoma;
6. ProGRP is a biomarker of SCLC;
7. SCCA and mucin are biomarkers of adenosquamous carcinoma;
8. SST is a biomarker of large cell neuroendocrine carcinoma.

## 10. Conclusions

The great phenotypic diversity of each histological lung cancer type and the absence of highly specific and sensitive biomarkers make lung cancer diagnosis rather difficult. Panels of various biomarkers have been recently applied and are becoming more popular as this technique improves early lung cancer detection. In this review, we suggest the use of a panel consisting of eight tumor-associated biomarkers—CEA, CYFRA21-1, ProGRP, CEA, PSF3, MUC, SCCA, and SST—allow us to differentiate between each histological type of lung cancer and to define the metastasis rate. In addition to conventional biomarker discovery methods, aptamer-based detection of several lung cancer biomarkers, such as LMN and VIM, DEF, and TUB, could be helpful for accurate diagnostics.

## Figures and Tables

**Table 1:** Protein biomarkers of lung cancer defined using proteomic studies.

Lung Cancer Type	Protein Biomarkers of Lung Cancer	Reference
NSCLC, SCLC	AGER, C10orf116, ADD2, PRX, LAMB3, SYNM, SPTA1, ANK1, HBE1, HBG1, CA1, TNXB, MMRN2, HBA1, CAV1, HBB, COL6A6, C1orf198, CLIC2, SDPR, EHD2, APOA2, NDUFB7, PRKCDBP, LAMA3, LBN	[33,34,35]
	ACT, 3 IGFBP3, L-PGDS	[35]
	SAA	[36]
	SAA, HAP, HGF	[36,37]
	TTR	[38,39]
	SAA, AAG1/2, CLU, SSA, AAG1, SAA, TTR	[6,35,37,40,41]
	APOA4, FIBA, LBN, SAA, CP, HP, TTR, KRT2A, GLT1B, CK1, AKT, MBL2, AAG1-2, FGA	[42]
	GSN, HP, FCN3, CNDP1	[43]
Lung adenocarcinoma	CALCA, CPS1, CHGB, IVL, AGR2, NASP, PFKP, THBS2, TXNDC17, PCSK1, CRABP2, ACBD3, DSG2, LRBA, STRAP, VGF, NOP2, LCN2, CKMT1B, AKR1B10, PCNA, CPD, PSME3, VIL1	[44,45,46,47]
Squamous lung cancer	SERPINB5, RPL5, PKP1, RPL10, AKR1B10, AKR1C1, PCNA, RPS2, AKR1C3, THBS2, ACBD3, VSNL1, AHCY, IMMP10, PAK2, IVL, IARS, PSMD2, GBP5, MCM6, NDRG1, NOP58, S100A2, NRG1-2, CNDP1	[45,47]
	UCRP, CER, UPA, MT1-MMP, SFN, TF, ALB, S100A9, STMN, ENO, PLAU, IGFBP7, MMP14, THBS1, TTR	[48]

NSCLC: non-small-cell lung carcinoma; SCLC: small-cell lung carcinoma.

**Table 2:** Conventional protein biomarkers of lung cancer.

№	Protein Biomarkers of Lung Cancer	Reference
1	CEACAM (Carcinoembryonic Antigen)	[5,34,38,40,43,55,56,57,58,59,60,61,62]
2	CYFRA21-1 (Cytokeratin-19 fragments)	[5,8,26,34,38,43,55,56,58,61,62,63,64,65,66,67]
3	CA125 (Cancer Antigen 125)	[68]
4	PKLK (Plasma kallikrein)	[51]
5	ProGRP (Pro-gastrin-releasingpeptide)	[17,56,69,70]
6	NSE (Neuron-specific enolase)	[56,61,70,71,72]
7	ТРА 6, 7, 8	[23,43]
8	NRG2, 100	[43]
9	CNDP	[43]
10	APOВ100	[43]
11	SCC (Squamous cell carcinoma antigen)	[73,74]
12	VEGF (Vascularendothelial growth factor)	[56]
13	EGFR (Epidermal Growth Factor)	[50]
14	PIK3CA, HER2, BRAF, ROS, RET, NRAS, MET, MEK1	[6,7]
15	HER2	[75]
17	C4.4A	[20]
18	PSF3	[76,77]
19	FAM83B	[54]
20	ECD, CTNNB , VIM, S100A4	[47]
21	S100A7	[32]
22	COX2	[78]
23	MUC1	[74,79]

**Table 3:** Aptamers for lung cancer diagnostics and therapy.

Aptamers	Target Cells	Protein Target	Application	Reference
Small cell lung cancer				
HCA12 HCC03 HCH07 HCH01	Cell lines: NCI–H69 NCI–H146 NCI–H128	Not determined	Formalin-fixed, Paraffin-embedded Tissue Array; Extraction and Detection with Aptamer Conjugated Magnetic/Fluorescent Nanoparticles using fluorescence microscopy and flow cytometry	[108]
16-1	SBC3 cell line	Not determined	Fluorescence microscopy and flow cytometry	[109]
Lung Adenocarcinoma				
EJ7 ADE2	H23 cell line H23, A549 cell line	Not determined	Flow cytometry	[110]
S13, S50	EGFR-transfected A549 cell line	EGFR	Antiproliferative activity	[111]
R50	A549 cells transfected with EGFR-GFP	NCL	Apoptosis induction	[112]
LC-17	Post-operative tissue	TUB	Aptahistochemical analyses of tissues Isolation of circulating tumor cells	[62,88]
LC-18,	Post-operative tissue	VIM, LMN	Aptahistochemical analyses of tissues Isolation of circulating tumor cells Electrochemical detection of protein biomarkers in human blood plasma	[62,88,103]
LC-224	Post-operative tissue	ACT methylated at position 73	Aptahistochemical analyses of tissues	[88]
LC-110	Post-operative tissue	CLU H2B	Isolation of circulating tumor cells	[62]
LC-183	Post-operative tissue	CTSD	Isolation of circulating tumor cells Inhibition of growth of primary cancer cell cultures	[62,113]
MA3	Сell lines: A549, MCF-7	MUC1	Targeted delivery of doxorubicin	[114]
MUC-1 aptamer	A549 cell line	MUC1	Targeted delivery of plasmid DNA	[115]
GL21.T	A549 (Axl+) cell line	AXL	Aptamer used as carriers for cell-targeted delivery of a miRNA with tumor suppressor function, let-7g; miR-212	[116,117],
Other				
aptNCL	CL1-5 cell line	NC L	Targeted delivery of siRNA chimeras	[118]
AS1411	Multiple cancer cell types	NC L	PET imaging of lung cancer with Cu-64 labeled aptamer	[119]
S1, S6, S11e, S15	NSLC	Not determined		[120]

**Table 4:** Comparative levels of lung cancer biomarkers in blood plasma of patients with non-small-cell lung carcinoma (NSCLC) and small-cell lung carcinoma (SCLC) and healthy people.

Tumor-Associated Protein	NSCLC	SCLC	Normal
LDH	525.079 ± 24.817 ng mL^−1^ [134]	209.880 ± 161.322 ng mL^−1^ [134]	<245 ng mL^−1^ [134]
CRP	25.079 ± 24.817 ng mL^−1^ [134]	14.935 ± 21.078 ng mL^−1^ [134]	<8 ng mL^−1^ [134]
CEA	51.493 ± 77.529 ng mL^−1^ [134] 78.5 ng mL^−1^ [23] ≥ 100 ng mL^−1^ [65]	25.074 ± 40.957 [134]	<5.0 ng mL^−1^ 5.0 ng mL^−1^ [23,61] <20.9 ng mL^−1^ 6.5 ng mL^−1^ [66]
NSE	13.638 ± 5.571 ng mL^−1^ [134] >6.4 ng mL^−1^ [19] 5–35 ng mL^−1^ 17.95 ng mL^−1^ [61] 0–170 ng mL^−1^ [23]	62.972 ± 63.012 [134] 50.8 ng mL^−1^ [61] 15–173 ng mL^−1^ [23]	15.7–17.1 ng mL^−1^ 15.2 ng mL^−1^ 13 ng mL^−1^ [65]
CYFRA21-1	12.447 ± 15.814 ng mL^−1^ [134] 81.7 ng mL^−1^ [23]	6.418 ± 9.567 ng mL^−1^ [134]	<3.3 ng mL^−1^ [134] 3.3 ng mL^−1^ [35] 3.3 ng mL^−1^ [61,65] 0.5 ng mL^−1^ [65] 2.0 ng mL^−1^ [23]
SCCA	0.22–3.79 ng mL^−1^ [61] 0.5–1.7 >2 ng mL^−1^ [135]	0.15 ng mL^−1^ [61]	1.5 ng mL^−1^ [23]
TPS	0–3842 ng mL^−1^ [136]	12.5–773 ng mL^−1^ [23]	34.9 ng mL^−1^ UL^−1^ [23]
ProGRP	<35 pg mL^−1^ [22]	>200 pg mL^−1^ [22]	<35 pg mL^−1^ [22]

**Table 5:** Comparative levels of well-known lung cancer biomarkers in blood plasma of patients with adenocarcinoma and squamous lung cancer and healthy people.

Tumor-Associated Protein	Adenocarcinoma	Squamous Carcinoma	Normal
CEA	30.76 ng mL^−1^ [61] 0.6–588 ng mL^−1^ [23] 3.5–11.1 ng mL^−1^ [66]	4.49 ng mL^−1^ [134] 0.8–587 ng mL^−1^ [23]	<5.0 ng mL^−1^ 5.0 ng mL^−1^ [23,61] <20.9 ng mL^−1^ 6.5 ng mL^−1^ [66]
NSE	17.95 ng mL^−1^ [134]	16.83 ng mL^−1^ [134]	15.7–17.1 ng mL^−1^ 15.2 ng mL^−1^ 13 ng mL^−1^ [23]
CYFRA21-1	4.00 ng mL^−1^ [61] 5.79 ± 6.75 ng mL^−1^ [63] 1.3–4.4 ng mL^−1^ [66]	10.34 ng mL^−1^ [61]	<3.3 [134] 3.3 ng mL^−1^ [23,61] 0.5 ng mL^−1^ [23] 2.0 ng mL^−1^ [66]
SCCA	0.22 ng mL^−1^ [61] 0.5–1.7 >2 ng mL^−1^ [66]	3.79 ng mL^−1^ [61]	1.5 ng mL^−1^ [66]
TPS	10–3842 ng mL^−1^ [23]	0–3000 ng mL^−1^ [23]	34.9 ng mL^−1^ [23]

**Table 6:** A panel of biomarkers specific for SCLC, adenocarcinoma, squamous lung cancer, and large cell lung cancer.

Biomarker	CEA ngmL^−1^	NSE ngmL^−1^	ProGRP pgmL^−1^	PSF3	CYFRA21-1 ngmL^−1^	SCCA ngmL^−1^
Small Cell CA	25.07 ± 41.1	50.8–173	>200	normal	6.42 ± 9.57	0.15
AdenoCA	0.6–588	17.95	~35	overexpression	1.3–5.79	0.22–2.0
SquamousCA	0.8–587	16.83	~35	normal	10.34	3.79
Large Cell CA	51.5–100	4.6–17.95	~35	normal	1.3–5.79	0.22–2.0
Healthy	5–20.9	13–17.1	~35	normal	0.5–1.3	1.5
